# Sodium-glucose cotranspor ter 2 (SGLT2) inhibitors in nephrolithiasis: should we “gliflozin” patients with kidney stone disease?

**DOI:** 10.1590/2175-8239-JBN-2023-0146en

**Published:** 2024-02-12

**Authors:** Mauricio de Carvalho, Ita Pfeferman Heilberg

**Affiliations:** 1 Pontifícia Universidade Católica do Paraná, Faculdade de Medicina, Curitiba, Paraná, Brazil.; 2 Universidade Federal do Paraná, Departamento de Clínica Médica, Curitiba, Paraná, Brazil.; 3 Universidade Federal de São Paulo, Escola Paulista de Medicina, São Paulo, São Paulo, Brazil.

**Keywords:** Nephrolithiasis, Kidney Calculi, Sodium-Glucose Transporter 2 Inhibitors

## Abstract

The prevalence of nephrolithiasis is increasing worldwide. Despite advances in understanding the pathogenesis of lithiasis, few studies have demonstrated that specific clinical interventions reduce the recurrence of nephrolithiasis. The aim of this review is to analyze the current data and potential effects of iSGLT2 in lithogenesis and try to answer the question: Should we also “gliflozin” our patients with kidney stone disease?

## The iSGLT2 Saga

**Figure 1:**
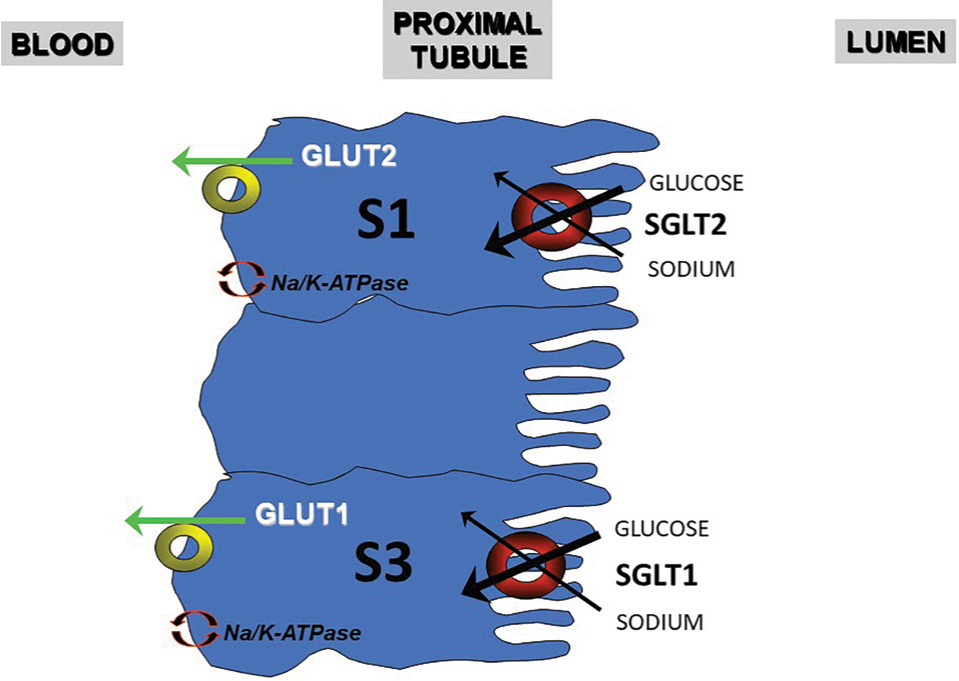
SGLT2 is located at the beginning (S1) of the proximal tubule, and it is responsible for 80–90% of the filtered glucose reabsorption. SGLT1 is on the most distal portion of the proximal tubule (S2/S3) and it is responsible for reabsorbing the 10–20% remaining of the filtered glucose.

Approximately 180g of glucose is filtered daily by the kidney on average. Around 90% of filtered glucose is reabsorbed in the proximal convoluted tubule^1^. Glucose enters the cell by an active process mediated by sodium, through sodium glucose cotransporters (SGLT). At the beginning of the proximal convoluted tubule, SGLT2 (encoded by the SLC5A2 gene) is responsible for most of the glucose reabsorption, which is completed in the straight part by SGLT1 (SLC5A1 gene), also expressed in enterocytes. In the basolateral membrane, glucose is transported by a facilitated diffusion process through the GLUT2 glucose transporter (SLC2A2 gene), from the GLUT^2,3^ family of transporters (Figure 1).

In 1996, Japanese researchers developed gliflozins, analogues of phlorizin, a compound from apple tree bark^4^. They demonstrated the ability of these substances to inhibit SGLT2 (iSGLT2) channels and cause glycosuria, opening up the prospect of use in individuals with diabetes mellitus (DM). Since then, several gliflozins have been synthesized (empagliflozin, canagliflozin, dapagliflozin, among others). It is worth mentioning that, in 2008, after a meta-analysis with rosiglitazone (a hypoglycemic agent from the thiazolidinedione class) demonstrated an increased risk of myocardial infarction, the FDA began to require cardiovascular safety studies for antidiabetic medications^5^. These trials analyzed safety in terms of major cardiovascular events (mortality due to cardiovascular causes, non-fatal AMI and non-fatal stroke). In this context, in 2015 the first data from the EMPAREG OUTCOME study were published. This, when analyzing 7,020 patients, demonstrated that empagliflozin reduced blood glucose and cardiovascular risk by up to 38% in patients with DM2 and a history of cardiovascular diseases when compared to placebo^6^. In 2019 and 2021, DAPA-CKD and CREDENCE (which only involved diabetics) and, in 2022, the EMPA-KIDNEY demonstrated that iSGLT2 improves renal outcomes and delays the progression of CKD, regardless of the patient having DM and in all KDIGO^7,8^ risk categories.

Although its mechanism of action is not fully elucidated, the beneficial effects of iSGLT2 are pleiomorphic, detected in clinical and pre-clinical studies, and include improvements in blood glucose, weight loss, reduction in blood pressure, improvements in cardiovascular and renal risk factors, reduced risk of stroke and even reduced risk of cancer (mechanisms unclear, exploratory analysis), detected in some studies^9^.

Current indications for iSGLT2 include congestive heart failure (regardless of ejection fraction), glycemic or metabolic risk control in patients with DM, reduction of cardiovascular risk in DM, diabetic and non-diabetic kidney disease, such as IgA nephropathy^10^.

The most common adverse effects of iSGLT2 include genital mycotic infections, hypoglycemia (especially with concomitant use of insulin or insulin secretagogues in type 1 diabetics) and extracellular volume depletion. And more rarely, euglycemic diabetic ketoacidosis, lower limb amputations (detected exclusively in the CANVAS study), bladder cancer, bone fractures, urinary infection and acute kidney injury^11^.

There are currently several ongoing studies seeking to analyze the uses of iSGLT2 in situations such as correction of hypomagnesemia; stimulation of erythropoiesis in anemia; increased free water clearance in hyponatremia; beneficial effects on cardiorenal syndrome; and decreased cardiovascular and renal morbidity and mortality in patients undergoing kidney transplantation^12^.

The aim of this review is to analyze the current data and potential effects of iSGLT2 in lithiasis disease and try to answer the question: Should we also “gliflozin” our patients with kidney stone disease?

## Evidence 1: Experimental Models

In 2011, Ly et al. developed a mouse model carrying a mutation in the SLC5a2 gene, which resulted in SGLT2^13^ function loss. The phenotype of this model (*Sweet pee*) was similar to patients with mutations in this gene in familial renal glycosuria. After diabetes induction, when compared to wild-type mice, those homozygous for the SGLT2 mutation showed a higher rate of glycosuria, polydipsia and increased urinary volume. When analyzing the amount of urine eliminated per 25g of body weight, there was a higher excretion of calcium, sodium, phosphorus and magnesium^13^. The authors concluded that this model of SGLT2 inhibition improved glycemia. However, the risk of infection, malnutrition, depletion and mortality in animal studied increased.

In 2023, Anan et al.^14^ induced the formation of calcium oxalate stones in Sprague-Dawley rats using ethylene glycol and alfacalcidol. Rats that used iSGLT2 (phlorizin) had reduced stone formation and the expression of KIM-1 and osteopontin. There was no change in water intake or urinary volume in these animals. There was inflammation suppression and decreased expression of macrophages, suggesting an anti-inflammatory role of iSGLT2 in lithogenesis.

## Evidence 2: an “Early” Meta-Analysis

In 2019, a few years after the first clinical trials with iSGLT2, Cosentino et al.^15^ published a post hoc analysis as an addendum to a previous meta-analysis on iSGLT2. They analyzed 27 studies lasting at least 52 weeks in diabetic patients, comparing iSGLT2 with placebo or other antidiabetic medications. The primary objective was the incidence of nephrolithiasis, reported by the investigators as a serious adverse event.

Of the 27 studies included, totaling 32,931 patients, 16 reported at least one case of nephrolithiasis (62 in the iSGLT2 and 44 in the control group). There was no association between iSGLT2 and nephrolithiasis (OR 0.85 [0.57–1.26])^15^.

Limitations of this study include the fact that nephrolithiasis was not among the pre-specified outcomes. Therefore, it is possible that some of the events were not classified as serious adverse events, raising the possibility of underreporting. Furthermore, small differences in the risk of nephrolithiasis between treatment groups may have gone unnoticed due to the small number of events (total of 106 cases of nephrolithiasis).

## Evidence 3: a Whole-Country Observational Cohort

Kristensen et al.^16^ used data from Danish health records from 2012 to 2018 to study diabetics aged ≥40 years using iSGLT2 or glucagon-like peptide-1 receptor agonists (GLP1 RAs). The patients were followed from the beginning of treatment until the diagnosis of nephrolithiasis, death, emigration or end of the study. After a 2-year follow-up, 12,325 individuals using iSGLT2 had a relative risk rate of nephrolithiasis (hazard ratio, HR) of 0.51 (0.37–0.71) compared to the same number of patients using GLP1RA. The incidence of nephrolithiasis per 1,000 patient-years was 2.0 (1.6–2.6) in the iSGLT2 group and 4.0 (3.3–4.8) in the GLP1RA^16^ group.

In addition to using active comparators (GLP1RA and repeat analysis with dipeptidyl peptidase-4 inhibitors, iDPP4), the authors considered baseline differences in patient characteristics using propensity scores. However, they could not fully exclude residual confounding factors due to unmeasured covariates, such as weight loss associated with GLP1RA use. Furthermore, they did not have data on lifestyle, including diet, kidney function or 24-hour urine output.

## Evidence 4: a Post hoc Analysis of 24-Hour Urine from Healthy Volunteers

In a post hoc analysis of a previous study, 45 volunteers with normal renal function, without kidney stones detected on ultrasound, were studied^17^. The group consisted of 27 men, 18 women, mean age of 33.4 ± 0.99 years and BMI of 28.2 ± 0.1 kg/m^2^. Of these, 40 completed the study and received 10 mg empagliflozin (n = 27) or placebo (n = 13) for 4 weeks.

In the empagliflozin group, there was a 45% increase in citraturia and a reduction in urinary pH, both during the day (6.4 ± 0.9 vs. 5.9 ± 0.9, p = 0.004) and at night (6.0 ± 0. 8 vs. 5.6 ± 0.6, p = 0.02). The authors concluded that empagliflozin reduced urinary supersaturation (SS) for calcium phosphate, an effect mediated by increased citraturia and decreased urinary pH^17^.

It should be noted that, in contrast to the decrease in SS for calcium phosphate, there was an increase in SS for uric acid. The group treated with empagliflozin had a slight, non-significant increase in uricosuria. However, there was a marked reduction in urinary pH, the most important factor for the formation of uric acid stones. There was no change in calcium oxalate SS.

## Evidence 5: a Post Hoc Analysis of Multiple Studies of Empagliflozin for Patients with T2DM

A total of 15,081 individuals were analyzed, from 20 placebo-controlled, randomized, phase 1 to 4 studies^18^. Patients had DM, using empagliflozin (n = 10,177) or placebo (n = 4,904). The average exposure to the drug was 543 days for placebo and 549 days for empagliflozin. Incident kidney stones were considered adverse events. Incidence rate ratios (IRR) and 95% confidence intervals per 100 patient years were calculated using the relative risk estimate, stratified by the study.

During follow-up, 183 patients experienced an episode of urolithiasis (placebo, 79; empagliflozin, 104), with annual incidence rates of 1.01 vs. 0.63 events/100 patient-years in the two groups, respectively. The IRR was 0.64 (95% CI, 0.48–0.86), in favor of empagliflozin, a reduction of approximately 40% in diabetics^18^.

Limitations of this study are common to previous studies and include the post hoc analysis and the fact that the presence of nephrolithiasis was based on adverse events reported by the investigators. Finally, 24-hour urine samples were not available for the SS analysis.

## Evidence 6: Analyses of Large Databases, with and Without DM

Using databases from two private healthcare insurance companies and Medicare (2013–2019), in an abstract published in Kidney Week 2022, Paik et al.^19^ found 102,275 pairs of adults with DM2 who used empagliflozin or iDPP4. They also analyzed 115,489 pairs who started using empagliflozin or a GLP1RA. The objective was to identify nephrolithiasis, diagnosed by ICD-10, in a hospital or outpatient setting. Relative risks (RR), rate differences (RD), and 95% confidence intervals (CI) were calculated, adjusted for 148 covariates.

During an 8-month follow-up, the risk of nephrolithiasis was lower in the empagliflozin group compared to the iDPP4 group (RR 0.72 [95% CI 0.67–0.78]; RD/1,000 person-years –6.2 [95% CI –7.6, –4.8]) and the group that used GLP1RA (HR 0.73 [95% CI 0.68–0.79]; RD/1,000 person-years –6, 0 [95% CI –7.4, –4.6])^19^.

Another study, using a large Japanese administrative database, divided diabetic patients (n = 1,538,198) according to iSGLT2^14^ prescription status. The study population consisted of patients aged ≥20 years with DM, and data were collected from January to December 2020. Patients with ICD-10 code = E10-E14 were defined as patients with DM, while those with ICD-10 = N20 were defined as lithiasis patients.

The prevalence of nephrolithiasis in men (n = 909,628) with DM was lower in the iSGLT2 group compared to the group not treated with iSGLT2 (2.28% vs. 2.54%, OR: 0.89, 95% CI [0.86–0.94]). However, the frequency of nephrolithiasis was not different between diabetic women (n = 628,570) treated with and without iSGLT2^14^.

The same authors, using the same database, also validated the study for patients without DM^20^. iSGLT2 prescription in male patients without DM was associated with a lower likelihood of urolithiasis (OR, 0.42; 95% CI, 0.35–0.51). In contrast, in non-diabetic women, iSGLT2 prescription was not associated with lower odds of urolithiasis (OR, 0.90; 95% CI, 0.68–1.19).

In addition to the previously mentioned limitations (post hoc analysis and nephrolithiasis analyzed as an adverse event), the authors recognize the lack of detailed information on the severity of DM and on compliance and duration of iSGLT2^20^ use.

## Verdict (?): a Randomized Clinical Trial (?)

W. Edwards Deming, known for his quality management theories, states that “Without data, you are just another person with an opinion.” Considering the studies presented in this review, we need a clinical trial with iSGLT2 that places nephrolithiasis as the primary objective (Table 1).

**Table 1:** Summary of the main results

Experimental model (*Sweet pee*): adverse results. 2019 negative Metanalysis for benefits: early, very few events. Danish retrospective cohort vs. GLP1a: RR – 0.51 favoring iSGLT2. 24h U from healthy volunteers: ↓SSCaP (↓pH; ↑citrate); ↑SSUA. Empagliflozin cohort in DM2 – IRR 0.64 vs. placebo. Large database analysis, with and without DM – RR and OR favoring the iSGLT2.

In 2022, the SWEETSTONE^21^ study protocol was published, aiming to evaluate the impact of empagliflozin on urinary SS in a randomized, double-blind, placebo-controlled, cross-over study. The inclusion criteria were age between 18–74 years, glycated hemoglobin <6.5%, history of one or more stones containing ≥80% calcium or ≥80% uric acid. The primary outcome was composite and includes changes in the SS of calcium oxalate, calcium phosphate and uric acid after treatment with empagliflozin^21^. The hypothesis is that empagliflozin would reduce urinary SS, and consequently the formation of new stones. The study was scheduled to be completed by the end of 2022^*^.

## Conclusions

Nephrolithiasis is a global healthcare problem in almost all developed and developing countries. Its prevalence has increased, with a high recurrence rate. Despite advances in understanding lithiasis disease, it is crucial to develop effective strategies to prevent nephrolithiasis, an unmet need. Although preliminary results with iSGLT2 are encouraging, this hypothesis needs to be tested in lithiasis patients, primarily aiming to reduce urinary stones. Pending the results of randomized clinical trials, one approach to be adopted at this time would be to associate gliflozins to the treatment of lithiasis patients with already established indications for these medications, such as in concomitant diabetic kidney disease, for instance.

## Footnotes

* According to the clinicalTrials.gov website, accessed on 12/04/2023, there were still no results posted.
