# Supplementary material for: Sodium-glucose cotranspor ter 2 (SGLT2) inhibitors in nephrolithiasis: should we “gliflozin” patients with kidney stone disease?
Source: J Bras Nefrol. 2024 Feb 12;46(3):e20230146. doi: 10.1590/2175-8239-JBN-2023-0146en (PMC11287977; doi:10.1590/2175-8239-JBN-2023-0146en)
Supplement: Translation

# Translation: Inibidores de cotransportadores sódio-glicose-2 (SGLT2) na nefrolitíase – devemos “gliflozinar” os litiásicos?

**DOI:** 10.1590/2175-8239-JBN-2023-0146pt

**Authors:** Mauricio de Carvalho, Ita Pfeferman Heilberg

**Affiliations:** 1 Pontifícia Universidade Católica do Paraná, Faculdade de Medicina, Curitiba, Paraná, Brasil.; 2 Universidade Federal do Paraná, Departamento de Clínica Médica, Curitiba, Paraná, Brasil.; 3 Universidade Federal de São Paulo, Escola Paulista de Medicina, São Paulo, São Paulo, Brasil.

**Keywords:** Nefrolitíase, Cálculos Renais, Inibidores do Transportador 2 de Sódio-Glicose

## Abstract

A prevalência da nefrolitíase está aumentando em todo o mundo. Apesar dos avanços na compreensão da patogênese da doença litiásica, poucos estudos demonstraram que intervenções clínicas específicas diminuem a recorrência da nefrolitíase. O objetivo desta revisão é analisar os dados atuais e efeitos potenciais dos iSGLT2 na doença litiásica e tentar responder à pergunta: devemos também “gliflozinar” os litiásicos?

## A SAGA DOS ISGLT2

Em média, aproximadamente 180g de glicose são filtrados diariamente pelo rim. Cerca de 90% da glicose filtrada é reabsorvida no túbulo contornado proximal^1^. A glicose entra na célula por um processo ativo mediado pelo sódio, através de cotransportadores de sódio e glicose (SGLT). No início do túbulo contornado proximal, o SGLT2 (codificado pelo gene SLC5A2) é responsável pela maior parte da reabsorção de glicose, que é completada na parte reta pelo SGLT1 (gene SLC5A1), também expresso nos enterócitos. Na membrana basolateral, a glicose é transportada por um processo de difusão facilitada através do transportador de glicose GLUT2 (gene SLC2A2), da família de transportadores GLUT^2,3^ (Figura 1).

**Figura 1:** O SGLT2 está localizado no início (S1) do túbulo proximal e é responsável pela reabsorção de 80–90% da glicose filtrada. O SGLT1 está localizado na parte mais distal do túbulo proximal (S2/S3) e é responsável pela reabsorção dos restantes 10–20% da glicose filtrada.

Em 1996, pesquisadores japoneses desenvolveram as gliflozinas, análogas da florizina, um composto proveniente da casca da macieira^4^. Demonstraram a capacidade destas substâncias de inibir os canais SGLT2 (iSGLT2) e provocar glicosuria, abrindo uma perspectiva de uso nos indivíduos portadores de diabetes mellitus (DM). Desde então, várias gliflozinas foram sintetizadas (empagliflozina, canagliflozina, dapagliflozina, entre outras). Vale ressaltar que em 2008 o FDA, após uma metanálise com a rosiglitazona (hipoglicemiante da classe das tiazolidinedionas) demonstrar aumento de risco de infarto do miocárdio, passou a exigir estudos de segurança cardiovascular para medicamentos antidiabéticos^5^. Estes ensaios analisaram a segurança em termos de eventos cardiovasculares maiores (mortalidade devida a causas cardiovasculares, IAM não fatal e AVC não fatal). Neste contexto, em 2015 foram publicados os primeiros dados do estudo EMPAREG OUTCOME. Este, ao analisar 7.020 pacientes, demonstrou que a empagliflozina reduziu a glicemia e o risco cardiovascular em até 38% em pacientes com DM2 e antecedentes de doenças cardiovasculares quando comparado com o placebo^6^. Em 2019 e 2021, o DAPA-CKD e o CREDENCE (que envolveram apenas diabéticos) e em 2022 o EMPA-KIDNEY, demonstraram que os iSGLT2 melhoram os desfechos renais e retardam a progressão da DRC, independentemente da presença de DM e em todas as categorias de risco do KDIGO^7,8^.

Apesar de seu mecanismo de ação não estar totalmente elucidado, os efeitos benéficos dos iSGLT2 são pleiomorfos, detectados em estudos clínicos e pré-clínicos e incluem melhora da glicemia, perda de peso, diminuição da pressão arterial, melhora nos fatores de risco cardiovasculares e renais, diminuição do risco de acidente vascular cerebral e até redução do risco de câncer (mecanismos não esclarecidos, análise exploratória), detectado em alguns estudos^9^.

Indicações atuais dos iSGLT2 incluem insuficiência cardíaca congestiva (independente da fração de ejeção), controle glicêmico ou do risco metabólico em pacientes com DM, redução do risco cardiovascular no DM, doença renal diabética e não diabética, como na nefropatia por IgA^10^.

Efeitos adversos mais comuns dos iSGLT2 incluem infecções micóticas genitais, hipoglicemia (principalmente com o uso concomitante de insulina ou secretagogos de insulina, em diabéticos tipo 1) e depleção do volume extracelular. E mais raramente, cetoacidose diabética euglicêmica, amputações de membros inferiores (detectada exclusivamente no estudo CANVAS), câncer de bexiga, fraturas ósseas, infecção urinária e injúria renal aguda^11^.

Atualmente, vários estudos em andamento procuram analisar usos dos iSGLT2 em situações como correção da hipomagnesemia; estímulo da eritropoiese na anemia; aumento da depuração de água livre na hiponatremia; efeitos benéficos na síndrome cardiorrenal; e diminuição de morbimortalidade cardiovascular e renal em pacientes submetidos a transplante renal^12^.

O objetivo desta revisão é analisar os dados atuais e efeitos potenciais dos iSGLT2 na doença litiásica e tentar responder à pergunta: devemos também “gliflozinar” os litiásicos?

## Evidência 01: Modelos Experimentais

Em 2011, Ly e colaboradores desenvolveram um modelo de camundongos portadores de mutação no gene SLC5a2, que resultava na perda da função do SGLT2^13^. O fenótipo deste modelo (*Sweet pee*) era similar aos pacientes com mutações nesse gene na glicosuria familiar renal. Após indução de diabetes, quando comparados aos camundongos selvagens, os homozigóticos para a mutação SGLT2 apresentaram maior taxa de glicosuria, polidipsia e aumento do volume urinário. Ao analisar a quantidade de urina eliminada por 25g de peso corporal, notou-se maior excreção de cálcio, sódio, fósforo e magnésio^13^. Os autores concluíram que este modelo de inibição do SGLT2 melhorou a glicemia. Entretanto, aumentou o risco de infecção, desnutrição, depleção e mortalidade nos animais estudados.

Em 2023, Anan et al.^14^ induziram a formação de cálculos de oxalato de cálcio em ratos Sprague-Dawley utilizando etilenoglicol e alfacalcidol. Os ratos que utilizaram iSGLT2 (florizina) diminuíram a formação de cálculos e a expressão de Kim 1 e osteopontina. Não houve alteração da ingestão hídrica e do volume urinário nestes animais. Notou-se supressão da inflamação e diminuição da expressão de macrófagos, sugerindo um papel anti-inflamatório dos iSGLT2 na litogênese.

## Evidência 02: uma Metanálise “Precoce”

Em 2019, poucos anos após os primeiros ensaios clínicos com os iSGLT2, Cosentino et al.^15^ publicaram uma análise *post hoc* como adendo a uma metanálise anterior sobre iSGLT2. Eles analisaram 27 estudos com duração de pelo menos 52 semanas, em pacientes diabéticos, comparando iSGLT2 com placebo ou com outras medicações antidiabéticas. O objetivo primário foi a incidência de nefrolitíase, relatada pelos investigadores como evento adverso grave.

Dos 27 trabalhos incluídos, totalizando 32931 pacientes, 16 relataram pelo menos um caso de nefrolitíase (62 nos iSGLT2 e 44 no grupo controle). Nenhuma associação entre os iSGLT2 e nefrolitíase foi observada (OR 0,85 [0,57–1,26])^15^.

As limitações deste estudo incluem o fato de que nefrolitíase não estava entre os desfechos pré-especificados. Portanto, é possível que alguns dos eventos não tenham sido classificados como eventos adversos graves, levantando a possibilidade de subnotificação. Além disso, pequenas diferenças no risco de nefrolitíase entre os grupos de tratamento podem ter passado despercebidas devido ao número pequeno de eventos (total de 106 casos de nefrolitíase).

## Evidência 3: uma Coorte Observacional de um País Inteiro

Kristensen et al.^16^ empregaram dados dos registros de saúde dinamarquês, de 2012 a 2018, para estudar diabéticos com idade ≥40 anos em uso de iSGLT2 ou agonistas do receptor de peptídeo-1 semelhante ao glucagon (GLP1 RAs). Os pacientes foram acompanhados desde o início do tratamento até o diagnóstico de nefrolitíase, óbito, emigração ou fim do estudo. Após um seguimento de 2 anos, 12.325 indivíduos em uso de iSGLT2 apresentaram uma taxa de risco relativo de nefrolitíase *(hazard ratio,* HR) de 0,51 (0,37–0,71) comparados ao mesmo número de pacientes utilizando GLP1RA. A incidência de nefrolitíase por 1000 pacientes-ano foi de 2,0 (1,6–2,6) no grupo iSGLT2 e de 4,0 (3,3–4,8) no grupo GLP1RA^16^.

Além de usar comparadores ativos (GLP1RA e repetição da análise com inibidores de dipeptidil-Peptidase 4, iDPP4), os autores consideraram as diferenças basais nas características dos pacientes por meio do escore de propensão. No entanto, não puderam excluir totalmente fatores de confusão residuais por covariáveis não mensuradas, como diminuição de peso associada ao uso de GLP1RA. Além disso, não possuíam dados sobre estilo de vida, incluindo dieta, função renal ou urina de 24 horas.

## Evidência 4: uma Análise *POST HOC* de urina de 24 horas de voluntários sadios

Em uma análise *post hoc* de um estudo prévio, 45 voluntários com função renal normal, sem cálculos renais detectados à ultrassonografia, foram estudados^17^. O grupo era formado por 27 homens, 18 mulheres, idade média de 33,4 ± 0,99 anos e IMC de 28,2 ± 0,1 kg/m^2^. Destes, 40 completaram o estudo e receberam 10mg de empagliflozina (n = 27) ou placebo (n = 13) por 4 semanas.

No grupo empagliflozina, houve aumento de 45% na citraturia e redução do pH urinário tanto diurno (6,4 ± 0,9 vs. 5,9 ± 0,9, p = 0,004) quanto noturno (6,0 ± 0,8 vs. 5,6 ± 0,6, p = 0,02). Os autores concluíram que a empagliflozina reduziu a supersaturação (SS) urinária para o fosfato de cálcio, efeito mediado pelo aumento da citraturia e diminuição do pH urinário^17^.

Deve-se notar que, em contrapartida à diminuição da SS para o fosfato de cálcio, houve aumento da SS para o ácido úrico. O grupo tratado com empagliflozina aumentou a uricosúria, de modo discreto, não significante. Entretanto, houve redução acentuada do pH urinário, o fator mais importante para a formação de cálculos de ácido úrico. Não houve alteração na SS do oxalato de cálcio.

## Evidência 5: uma Análise *POST HOC* de Vários Estudos com Empagliflozina Para Pacientes com DM2

Foram analisados 15081 indivíduos, provenientes de 20 estudos placebo-controlado, randomizados, de fase 1 a 4^18^. Os pacientes eram portadores de DM, em uso de empagliflozina (n = 10177) ou placebo (n = 4904). A média de exposição à droga foi de 543 dias para o placebo e 549 dias para a empagliflozina. Foram adjudicados cálculos renais incidentes como eventos adversos. As razões de taxas de incidência (IRR) e intervalos de confiança 95% por 100 pacientes/ano foram calculados utilizando a estimativa de risco relativo, estratificados por estudo.

Durante o acompanhamento, 183 pacientes apresentaram um episódio de urolitíase (placebo, 79; empagliflozina, 104), com taxas de incidência anual de 1,01 vs. 0,63 eventos/100 pacientes-ano nos dois grupos, respectivamente. A IRR foi de 0,64 (IC 95%, 0,48–0,86), a favor da empagliflozina, uma redução de aproximadamente 40% em diabéticos^18^.

As limitações deste estudo são comuns aos estudos anteriores e incluem a análise *post hoc* e o fato de que a presença de nefrolitíase foi baseada em eventos adversos relatados pelos investigadores. Por fim, amostras de urina de 24 horas não estavam disponíveis para análise da SS.

## Evidência 6: Análises de Grandes Bancos de Dados, com e sem DM

Utilizando banco de dados de dois planos de saúde privados e do Medicare (2013–2019), em um abstract publicado na Kidney Week 2022, Paik et al.^19^ identificaram 102.275 pares de adultos com DM2 que utilizaram empagliflozina ou iDPP4. Analisaram também 115.489 pares que iniciaram o uso de empagliflozina ou um GLP1RA. O objetivo foi identificar nefrolitíase, diagnosticada pelo CID-10, em ambiente hospitalar ou ambulatorial. Foram calculados os riscos relativos (HRs), diferenças de taxa (RD) e intervalos de confiança 95% (IC), ajustados para 148 covariáveis.

Durante um seguimento de 8 meses, o risco de nefrolitíase foi menor no grupo empagliflozina em comparação com o grupo que utilizou iDPP4 (HR 0,72 [IC 95% 0,67–0,78]; RD/1.000 pessoas-ano –6,2 [IC 95% –7,6, –4,8]) e o grupo que utilizou GLP1RA (HR 0,73 [IC 95% 0,68–0,79]; RD/1.000 pessoas-ano –6,0 [IC 95% –7,4, –4,6])^19^.

Outro trabalho, utilizando um grande banco de dados administrativo japonês, dividiu pacientes diabéticos (n = 1.538,198) de acordo com o status de prescrição de iSGLT2^14^. A população do estudo consistiu em pacientes com idade ≥20 anos com DM, e os dados foram coletados de janeiro a dezembro de 2020. Os pacientes com código CID-10 = E10–14 foram definidos como pacientes com DM, enquanto aqueles com CID-10 = N20 foram definidos como litiásicos.

A prevalência de nefrolitíase em homens (n = 909.628) com DM foi menor no grupo iSGLT2 comparada ao grupo não tratado com iSGLT2 (2,28% vs. 2,54%, OR: 0,89, IC95% [0,86–0,94]). No entanto, a frequência de nefrolitíase não foi diferente entre as mulheres (n = 628.570) diabéticas tratadas com e sem iSGLT2^14^.

Os mesmos autores, utilizando o mesmo banco de dados, validaram o estudo também para pacientes sem DM^20^. Em homens, a prescrição de iSGLT2 em pacientes sem DM foi associada a uma menor probabilidade de urolitíase (OR, 0,42; IC 95%, 0,35–0,51). Em contraste, em mulheres não diabéticas, a prescrição de iSGLT2 não foi associada a menor probabilidade de urolitíase (OR, 0,90; IC 95%, 0,68–1,19).

Além das limitações previamente citadas (análise *post hoc* e nefrolitíase analisada como evento adverso), os autores reconhecem a ausência de informações detalhadas sobre a gravidade do DM e sobre a adesão e tempo de utilização dos iSGLT2^20^.

## Veredito (?): Um Ensaio Clínico Randomizado (?)

W. Edwards Deming, conhecido por suas teorias de manejo de qualidade, afirma que “Sem dados, você é apenas mais uma pessoa com opinião.” Considerando os estudos apresentados nesta revisão, faz-se necessário um ensaio clínico com os iSGLT2 que coloque a nefrolitíase como objetivo primário (Tabela 1).

**Tabela 1:** Síntese dos principais resultados

Modelo experimental (*Sweet pee*): resultados adversos. Metanálise de 2019 negativa para benefícios: precoce, pouquíssimos eventos. Coorte retrospectiva dinamarquesa vs. GLP1a: HR – 0,51 a favor dos iSGLT2. U24 horas de voluntários sadios: ↓SSCaP (↓pH;↑citrato); ↑SSAU. Coorte empagliflozina em DM2 – IRR 0,64 vs. Placebo. Análises de grandes bancos de dados, com e sem DM – HR e OR favoráveis aos iSGLT2.

Em 2022 foi publicado o protocolo do estudo SWEETSTONE^21^. Este pretende avaliar o impacto da empagliflozina na SS urinária em um estudo randomizado, duplo cego, controlado por placebo, do tipo *cross-over*. Os critérios de inclusão são idade entre 18–74 anos, hemoglobina glicada <6,5%, história de um ou mais cálculos, que contenham ≥80% de cálcio ou ≥ 80% de ácido úrico. O desfecho primário é composto e inclui alteração na SS de oxalato de cálcio, fosfato de cálcio e do ácido úrico após o tratamento com empagliflozina^21^. A hipótese é que a empagliflozina diminuiria a SS urinária e por consequência a formação de novos cálculos. O estudo estava programado para ser concluído no final de 2022^*^.

## Conclusões

A nefrolitíase é um problema global de saúde em quase todos os países desenvolvidos e em desenvolvimento. Sua prevalência tem aumentado, com alta taxa de recorrência. Apesar dos avanços na compreensão da doença litiásica, é crucial desenvolver estratégias eficazes para prevenir a nefrolitíase, uma necessidade ainda não atendida. Embora os resultados preliminares com iSGLT2 sejam encorajadores, essa hipótese precisa ser testada em pacientes litiásicos, visando primariamente a redução de cálculos urinários. Na expectativa dos desfechos dos ensaios clínicos randomizados, uma conduta a ser adotada neste momento seria a de associar gliflozinas em litiásicos com indicações já estabelecidas destas medicações, como na concomitância de doença renal diabética, por exemplo.

## Footnotes

* De acordo com o site clinicalTrials.gov, acessado em 04/12/2023, ainda ão havia resultados postados.
